# TERT translocation as a Novel condition in Intrauterine Growth Restriction rats with early catch-up growth

**DOI:** 10.1371/journal.pone.0312221

**Published:** 2025-06-05

**Authors:** Guo-qian He, Yi-ling Dai, Zhuo Huang, Feng Ling, Ping Li

**Affiliations:** 1 Department of Pediatrics, West China Second University Hospital, Sichuan University, Chengdu, P.R. China; 2 Key Laboratory of Birth Defects and Related Diseases of Women and Children, Ministry of Education, West China Second University Hospital, Sichuan University, Chengdu, P.R. China; University of Life Sciences in Lublin, POLAND

## Abstract

Infants born with intrauterine growth restriction (IUGR) followed by postnatal rapid catch-up growth are prone to develop metabolic diseases later in life. However, its effects and underlying molecular mechanisms remain unclear. Male offspring from mothers fed a low-protein diet (maternal diet, 8% protein) were randomly assigned to one of the following groups: normal diet (RC group) and low-protein diet (RR group). Offspring were fed a normal-protein diet (maternal diet, 20% protein, control group). In our study, at birth, approximately 93.33% of the offspring fats from mothers fed a low-protein diet were born with IUGR. Following weaning, all IUGR groups showed catch-up growth. The RC groups showed accelerated and early postnatal catch-up growth and regained the same weight as the controls from 3 to 9 months. At 9 months of age, the RC group animals had shorter telomere length (TL) than the Control and RR groups and also showed higher oxidative stress levels and lipid levels. Furthermore, compared to the control group, there was increased mitochondrial translocation of telomerase reverse transcriptase (TERT) under conditions of elevated oxidative stress in the RC group. There was no significant difference in mtDNA content between the RC and control groups. Moreover, at 9 months of age, only in the RC group were liver and pancreas Sirt3 expression levels higher than in the Control and RR groups. These data indicate that IUGR with early and rapid catch-up growth is exposed to chronic oxidative stress and subsequently affects TL and TERT translocations. Chronic oxidative stress may promote the translocation of TERT from the nucleus to mitochondria and protect tissues from oxidative stress damage.

## Introduction

Intrauterine growth restriction (IUGR) is defined as the failure of a fetus to reach its growth potential, and is a common and significant complication during pregnancy [1]. Globally, IUGR occurs in 3%-10% of pregnancies and remains one of the leading causes of perinatal mortality and long-term morbidity [2]. Several causes may play a role in IUGR, including malnutrition, diabetes, and placental insufficiency [3,4]. The underlying causes of IUGR are complex. Maternal malnutrition during pregnancy is the most prevalent nongenetic or placental cause [5]. Numerous epidemiological studies have reported that IUGR is associated with metabolic syndrome, including lipid metabolic disorders, later in life [6]. IUGR is also an independent risk factor for childhood obesity. However, the effects and underlying mechanisms are not fully understood.

The “Developmental origins of health and diseases” (DOHaD) theory supposes that disadvantages in early life may be responsible for severe health problems later in life [7]. The catch-up growth (CUG) theory is regarded as the cause of metabolic diseases in individuals with IUGR [6,8]. CUG occurs in early postnatal life to accelerate toward the original pre-retardation growth curve, and has advantages in body growth. Different diets provided during pregnancy and after birth may be an important cause of metabolic disorders in adults with IUGR [9,10]. Studies of prospective human populations and animals have demonstrated that a nutritious diet accelerates the growth rate of infants with IUGR in the early stages of life and may have a higher risk of developing obesity and metabolic syndrome in later life [9,11,12]. When infants are fed food-restricted dams, rapid catch-up growth is delayed, preventing or diminishing adverse health consequences. However, another study showed that IUGR offspring fed restricted nutrition during lactation and fed a normal diet following weaning had a decreased body weight. Based on these results, the effect and mechanism of dietary nutrition on metabolic disorders in individuals with IUGR catch-up remain unclear.

It is well known that IUGR is associated with abnormal hypoxia/oxygenation conditions that interfere with molecular and bioenergetic processes [4,13]. A Decreased transfer of oxygen and nutrients to the fetus causes chronic hypoxia and a reduction in lipid levels [14]. Telomeres are nucleoprotein structures and have a significant function in protecting chromosomes from fusion and degradation [15,16]. Environmental factors, such as chronic oxidative stress, are related to accelerated telomere length (TL) shortening [17]. The accumulation of oxidative stress damage is less efficient in the telomeres. Moreover, TL has been linked to prenatal exposure to maternal diseases and nutritional disorders. Previous studies have demonstrated that rapid postnatal catch-up growth may result in increased telomere shortening in tissues [18–20]. In animal models, increasing nutrition to normal levels during lactation is associated with obesity and affects TL in IUGR rat offspring with rapid growth [18,21]. In addition, short telomeres were also found in small-for-gestational-age babies, who have an increased risk of developing metabolic diseases. However, the molecular mechanisms involved in this process remain unknown. Telomere length homeostasis requires telomerase, which is a cellular reverse transcriptase. Telomerase reverse transcriptase (TERT) is the catalytic subunit of telomerase and serves as a template for telomere extension by catalyzing the addition of short telomere repeats [22]. This may be due to downregulated telomerase activity or the redistribution of telomerase out of the nucleus in stressed tissues [23,24]. Research on the role of TERT in the metabolism of IUGR offspring that exhibit catch-up growth is rare. We hypothesized that chronic oxidative stress can lead to accelerated telomere shortening, which affects TERT and plays a role in IUGR during catch-up growth. The aim of this study was to evaluate telomere length in the adipose fat, pancreas, and liver, and to assess the expression of TERT in the liver and pancreas of IUGR rats undergoing a catch-up in body weight.

## Materials and methods

### IUGR rat models and dietary interventions

All experiments were performed in accordance with the Guide for the Care and Use of Laboratory Animals and were approved by the Research and Ethics Committee of West China Second Hospital of Sichuan University. This study was approved by the Institutional Animal Care and Use Committee of Sichuan University. An IUGR rat model was established according to a previously described protocol. Briefly, healthy female and male Sprague-Dawley (SD) rats (8 weeks old, 250–300 g) were purchased from Chengdu Dashuo Experimental Animal Co. Ltd. Female rats were mated with males overnight, and pregnancy was determined by the presence of spermatozoa in vaginal smears the following morning. We confirmed successful mating using vaginal spears, and evidence of sperm on the vaginal spear was considered day zero of gestation. During pregnancy, the first-time pregnant rats were randomly divided into a normal diet group (20% protein) and a low-protein diet group (8% protein, an isocaloric low-protein diet). Both diets were purchased from Xietong Co. Ltd. (Jiangsu, China). To avoid the influence of sex hormones on lipid metabolism, only male offspring were included in the final study.

Cross-fostering techniques were used to generate protein-restricted offspring during lactation (0–3 weeks) [25]. Following delivery, male rat offspring of the normal diet group were fed with normal foster mothers (maternal diet, 20% protein) during lactation and a normal diet (20% protein) after weaning (control group, n = 20). The IUGR neonatal male rats were divided into two groups with different dietary interventions. IUGR rats were fed with normal foster mothers (maternal diet, 20% protein) during lactation and a normal diet (20% protein) after weaning to create the experimental groups (recuperated offspring, RC group, n = 20). The other IUGR rats were fed a restricted diet (maternal diet, 8% protein) during lactation and a normal diet after weaning(20% protein) to create other experimental groups (RR group, n = 20). The three groups were weaned after 3 weeks and separated from their mothers. Dietary induction with a standard diet (20% protein) was completed at 9 months of age. The rats were caged individually and fed a fresh normal diet and water ad libitum in their home cages. The study protocol was reviewed and approved by the Ethics Committee of West China Second Hospital of Sichuan University (approval number: 2022084) and followed the principles of the Declaration of Helsinki.

### Body weights, BMI and tissues collection

Body weights (BWs) were recorded at day 0 (birth weight), 3 days, 7 days, 14 days, and 21 days, as well as per week from 21 days to 3 months, and per month from 3 to 9 months of age after birth. The birth weight of rat pups was recorded within 4 h of birth during the day or at 8:00 am the next morning if the birth occurred during the night. Body weight was recorded at 8:00 am on the postnatal days. Weight measurements were recorded to the nearest 0.1 kg, respectively. Based on the birth weights, IUGR pups were defined as the lower birth weights minus two standard deviations (SD) from the controls.

Body mass index (BMI) was calculated. To plot the growth rate, the weight SD Score (△SDS) was calculated. SDS=(measured body weight-average body weight of the same sex and age in the control group)/SD; △SDS = SDS of this time-SDS of the last time. At 9 months (36weeks) of age, which corresponds to approximately 17 years in humans. The rats were fasted overnight for 12 h prior to sampling. Blood was collected from the tail vein, kept standing for 45 min at room temperature, and then centrifuged for 15 min (3000 rpm, 4 °C). Plasma was separated using a micropipette, frozen in dry ice, and stored at -80 °C until analysis. At the study endpoint, rats were euthanized by gradual CO₂ inhalation in accordance with the AVMA Guidelines for Euthanasia (2020), and all efforts were made to minimize suffering. CO₂ was introduced into the chamber at a flow rate of 20% of the chamber volume per min. Death was confirmed by the absence of spontaneous breathing for 5 minutes, followed by secondary physical confirmation via cervical dislocation. The pancreas and liver were removed completely and frozen at -80 °C for later use.

### Blood lipid biomarkers analysis

Serum total cholesterol (TC), triglyceride (TG), high-density lipoprotein cholesterol (HDL-C), and low-density lipoprotein cholesterol (LDL-C) levels were measured using an automatic biochemical analyzer. All analyses were performed at the Laboratory of the West China Second Hospital of Sichuan University.

### Telomere length analysis

Relative TL was determined using quantitative real-time PCR (qRT-PCR). The average TL was calculated by the ΔCt method of the reference gene degree ratio (T/S ratio), which is the amplification of the telomere repeat region (T) relative to the amplification of a single-copy housekeeping gene (S), 36B4. Samples were run in triplicate in 96-well plates. A standard curve is composed of the reference DNA [26–28]. To ensure that only our targeted amplicons were amplified, dissociation melting curves were obtained for each sample. In cases where the standard deviation (SD) between triplicate samples was > 0.2, the sample was removed and re-run on the next plate. Additionally, samples with a coefficient of variance (CoV) > 0.15 were also removed and re-run. Each sample was replicated (in triplicate) on a separate plate to ensure that the T/S values were reliable. The average of these two independent T/S values is reported for each sample. In cases where the CoV between the two T/S values was > 0.15, the sample was re-run, and the average between the two most similar runs was reported.

### Evaluation of oxidative stress

The pathophysiology of IUGR is characterized by the deregulated production of reactive oxygen species (ROS). Malondialdehyde (MDA) levels, as an increased protein carbonyl content, were detected in adipose, pancreatic, and liver tissues in this study. 8-Isoprostaglandin F2a (8-iso-PGF2a) is a promising oxidative stress marker and was detected in the adipose tissue, pancreas, and liver using the commercially available Direct 8-iso-PGF2a ELISA kit (Abcam, Cambridge, UK). True reflection of both free and esterified isoprostane was measured following the manufacturer’s instructions. Lipid peroxidation was measured in duplicate as an indicator of oxidative damage to lipid membranes using the assay by measurement of s according to the manufacturer’s instructions.

### Immunofluorescence

Postmortem, the liver and pancreas were removed, snap-frozen in liquid nitrogen, and stored at -80◦C for further analysis. Immunofluorescence experiments were performed on liver and pancreas sections (3 µm). The sections were fixed with the immunofluorescence fixative for 20 min, followed by blocking with bovine serum albumin at room temperature for 1h. The cells were then incubated with primary antibodies, anti-TERT overnight at 4°C, and secondary anti-rabbit Alexa fluor-546-conjugated antibodies at room temperature for 2h. Nuclei were counterstained with DAPI (1µg/mL). The cells were observed and analyzed using a confocal microscope (Zeiss LSM780).

### Subcellular fractionation and Western blotting

Mitochondrial fractions were isolated from the pancreas and liver tissues using the Mitochondria Isolation Kit (Thermo Scientific), according to the manufacturer’s instructions. Nuclear fractions were extracted using the Nuclear/Cytosol Fractionation Kit. Protein concentrations were determined using the BCA Protein Concentration Determination Kit. Western blotting was performed to confirm that the subcellular fractions were pure and uncontaminated and to investigate the subcellular localization of TERT. Briefly, the protein concentration was measured using a BCA protein assay kit and separated by 10% SDS-PAGE. The separated proteins were subsequently transferred to polyvinylidene fluoride (PVDF) membranes and blocked with 5% skim milk. Membranes were then incubated with primary antibodies against TERT (Abcam, Cambridge, UK), COX-IV (Abcam, Cambridge, UK), and the cell nuclear marker Histone-H3 (Proteintech, Chicago, USA) overnight at 4°C. The membranes were washed with TBST three times and incubated with a secondary antibody (1:5000) for 1 h at room temperature. Immunoreactivity was detected using enhanced chemiluminescence (Bio-Rad) and visualized using an inverted fluorescent microscope (CKX41,Olympus,BA400Digatial). COX IV was used as an internal loading control to measure mitochondrial protein expression.

### Quantification of mtDNA content by real-time PCR

Total DNA was extracted from the pancreas and liver using the QIAamp DNA isolation kit. After quantification and adjustment of each genomic DNA concentration using Varioskan Flash (Thermo Scientific), samples were stored in separate aliquots at -80°C until analysis. mtDNA copy numbers were determined using real-time quantitative polymerase chain reaction (RT-PCR), according to the manufacturer’s instructions. GADPH was used as a nuclear target to quantify nuclear DNA and normalize the amount of mtDNA. All samples were run in triplicate for each gene, and the data were analyzed using the comparative Ct method, where Ct is the cycle number at which the instrument first detected fluorescence above background noise. The ∆ cycle threshold (∆ Ct) values of each sample were obtained by subtracting the values for the reference gene from the sample Ct, thus normalizing to nuclear DNA. For each experimental sample, 2- ∆ Ct was calculated and the data are presented as relative quantification. Melting curve analysis and electrophoresis were used to analyze the reaction specificity. The primer sequences were as follows: telomere, forward, 5’-CGGTTTGTTTGGGTTTGGGTTTGG-GTTTGGGTTTGGGTT-3’ and reverse, 5’-GGCTTGCCTTACCCTTACCCTTACCCTTACCCTTACCCT-3’; 36B4,forward, 5’-CAGCAAGTGGGAAGGTGTAATCC-3’ and reverse, 5’-CCCATTCTATCATCAACGGGTACAA-3’; and D-loopHV1, forward, 5’-GATTTGGGTACCACCCAAGTATTG-3’ and reverse, 5’-AATATTCATGGTGGCTGGCATGTA-3.’

### Immunohistochemistry

Liver and pancreas paraffin sections were layered with the primary antibody (1:200; rabbit anti-rat Sirtuin3 antibody; AB5583; Chemicon, Temecula, CA, USA) and incubated at 4°C overnight. After the addition of the secondary antibody, the staining procedures were carried out using peroxidase detection methods, as described previously. Each slide was counterstained with hematoxylin. Ten random visual fields were quantified per section (400 × magnification). The stained area was expressed as a percentage of the total medial area.

### Statistical analysis

Quantitative data are presented as mean± standard error of the mean (mean ± SEM). Student’s t-test or two-way analysis of variance (ANOVA) were used to analyze the differences between groups, as appropriate. Data were analyzed using SPSS software (version 19.0, Chicago, IL, USA). Statistical significance was set at p < 0.05. All experiments were performed in triplicates.

## Results

### IUGR-Induced lower body weight at Birth

The weights of the animals were determined after birth to identify the animals that had suffered IUGR. In this study, IUGR pups were produced by female rats that were fed a low protein diet (maternal diet, 8% protein) during gestation, and the average birth weights of the IUGR rat pups were significantly lower than those of the control group fed a normal diet (20% protein) (5.05 ± 0.33 g vs. 7.45 ± 0.80 g, P < 0.05). The incidence of IUGR pups was approximately 93.33% in this study, demonstrating that the IUGR model was successfully established.

### Weight growth trajectory in different groups of rats

**Fig 1:**
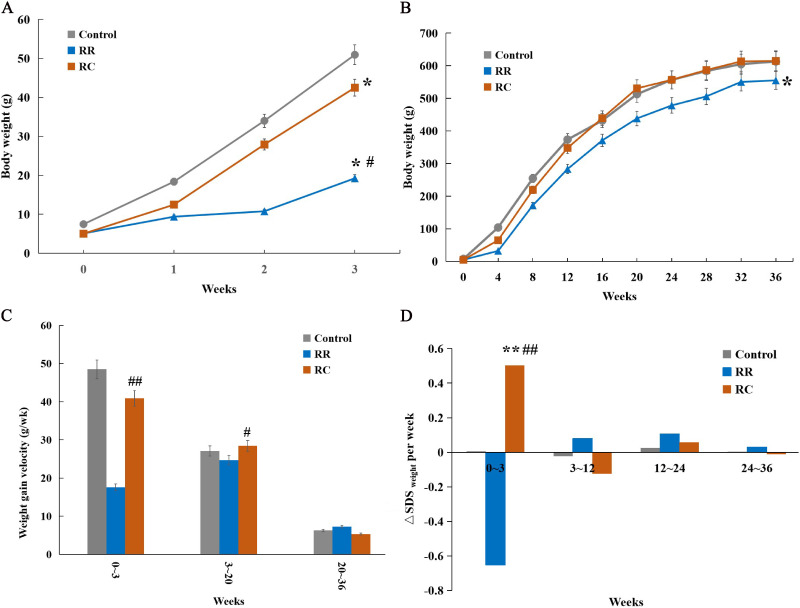
RC group showed an early catch-up growth. **A.** Body weights in different groups of rats during lactation (0-3 weeks). The RC group showed steeper growth slopes compared with the control group, whereas the RR group exhibited gentler growth than the control group. **B**. Body weights of different groups of rats from birth to 36 weeks. The RC group exhibited steeper growth slopes than did the control group. **C**. Body weight gain velocity in different groups of rats during lactation and after weaning. The RC group showed significant body weight gain during lactation compared to the RR group. **D**.SDSweight in different groups of rats during lactation and after weaning. The data on △SDSweight was higher in RC group during lactation compared with the Control group and RR group.Control, non-IUGR rats receiving a normal diet during lactation and after weaning; RC, IUGR rats cross-fostering to a normal nutrition dam during lactation and receiving a normal diet after weaning;RR, IUGR rats receiving a low-protein diet during lactation and a normal diet after weaning;IUGR, intrauterine growth retardation.△SDSweight,the weight SD Score.*p < 0.05 vs Control;^*#*^p < 0.05 vs RR;**p < 0.01 vs Control.##p < 0.01 vs RR.

To further analyze the effects of different dietary patterns during lactation and after weaning on BWs outcomes, the BWs of IUGR offspring was recorded and compared. There was no significant difference in the mean number of pups per nest or perinatal mortality between the groups. During the lactation period (0–3 weeks), the BWs in the RC and RR groups was lower than that in the control group (Fig 1A). The offspring in the RC group remained significantly heavier than those in the RR group throughout the lactation period. After weaning, all the rats were fed a standard diet (20% protein). The RC group exhibited steeper BWs growth slopes. There was no difference in body weight between the control and RC groups from 16 to 9 months of age. However, the BWs of the RR group were significantly lower at all time points than those of the control and RC groups (Fig 1B). The rate of weight gain was significantly higher in RC than in RR from the end of the first postnatal week to postnatal week 20 (Fig 1C). During lactation, the body weight gain velocity was approximately 2.3 times faster in the RC group than the RR group (40.83 ± 10.81g/wk vs. 17.55 ± 5.79 g/wk, P < 0.001) (Fig 1C). By comparison, △SDSweight in the RC group were significantly higher than those in the Control group (0.64 ± 0.12 per week vs. -0.001 ± 0.05 per week, P < 0.001) and the RR group (0.64 ± 0.12 per week vs. -0.42 ± 0.06 per week, P < 0.001) during the lactation period (0–3 weeks) (Fig 1D).

In addition, the pattern of weight gain differed among the three groups. Based on the mean and SD of the BWs, catch-up growth was defined as the BWs between the ”mean±SD” of the BWs of the Control group with the same sex and age. The RC group offspring showed early postnatal catch-up growth and were significantly heavier than the RR group from the end of the first postnatal week. After four weeks, the rats in the RC group exhibited accelerated catch-up growth. In contrast, the RR group exhibited gentler growth than the control group, and had incomplete catch-up growth during lactation and weaning. Thus, the offspring of the maternal interventions demonstrated early and accelerated postnatal catch-up growth that occurred much earlier in the RC group.

### BMI in different groups of rats

**Fig 2:**
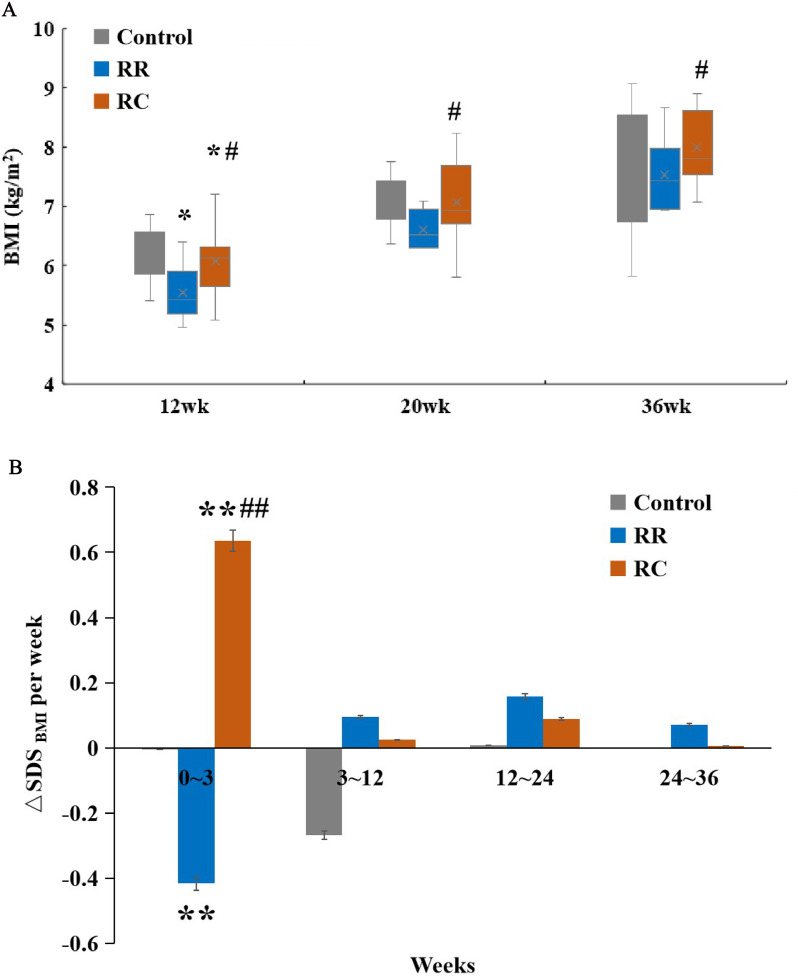
BMI and △SDS_BMI_ of rats significantly increased in RC group. **A.** BMI in different groups of rats after weaning: Compared to the RR group, the BMI of the RC group was significantly increased.**B.** SDS_BMI_ in different groups of rats during lactation and after weaning. During lactation, the RC group showed significant △SDS_BMI_ during lactation compared with the RR group.*Control, non-IUGR rats receiving a normal diet during lactation and after weaning; RC, IUGR rats cross-fostering to a normal nutrition dam during lactation and receiving a normal diet after weaning;RR, IUGR rats receiving a low-protein diet during lactation and a normal diet after weaning;IUGR, intrauterine growth retardation.△SDS*_*BMI*_, the BMI SD Score.*p < 0.05 vs Control;#p < 0.05 vs RR;**p < 0.01 vs Control.##p < 0.01 vs RR.

BMI data on BMI was also assessed at 12, 20, and 9 months of age. BMI was higher in the control group than in the RC or RR groups at 12 weeks of age. Over the subsequent weeks to 9 months, there was no statistically significant difference between the RC and control groups (Fig 2A). However, the BMI data were also consistently higher in the RC group than in the RR group at 12 and 20 weeks of age, which was underscored by the analysis of the BMI SD Score. The data on △SDSBMI was significantly higher in the RC group than the Control group (0.50 ± 0.15 per week vs. 0.01 ± 0.02 per week, P < 0.001) and the RR group (0.50 ± 0.15 per week vs. -0.65 ± 0.10 per week, P < 0.001) (Fig 2B). Thus, these tendencies for differences in BMI across the three groups were higher in the RC group.

### Early and accelerated catch-up growth accelerated Telomere shortening

At 3 months of age, there were no significant differences in the TL of the pancreatic tissues between the RR and control groups (P > 0.05, Table 1). Regarding telomere length, the differences in the liver tissues were also not significant (P > 0.05). At 3 months of age, the RC group showed catch-up growth and regained weight, but showed no differences in the TL of pancreatic and liver tissues compared to controls. Maternal diet had no significant effect on the liver and pancreatic TL.

**Table 1:** Comparison of relative Telomere Length (TL) by T/S among different IUGR groups.

		Control	RR	RC	*P*
Pancreatic tissue	3 mo.	2.150 (0.935 ~ 2.801)	1.982 (0.628 ~ 2.490)	1.807 (0.790 ~ 2.135)	0.647
	9 mo.	1.932 (1.209 ~ 3.095)	1.715 (0.837 ~ 2.602)	1.259(0.910 ~ 2.071)	0.009
Liver tissue	3 mo.	2.719 (0.817 ~ 3.924)	2.260 (1.42 ~ 3.917)	2.013 (1.01 ~ 3.611)	0.091
	9 mo.	2.366 (1.505 ~ 3.758)	1.975 (0.728 ~ 2.631)	1.407 (0.850 ~ 1.882)	0.012

However, by 9 months of age, the weight of the RC offspring was similar to that of the control group. Significantly shorter telomeres in the liver and pancreatic tissues were observed in the RC group than in the control group (P < 0.05, Table 1). In contrast, the RR group offspring still maintained a weight lower than that of the control group and showed a TL similar to that of the control group. Early and accelerated catch-up growth led to shorter TL at 9 months. To further investigate the role and underlying mechanisms of shorter TL, we chose this time period for the next study.

### Early and accelerated catch-up growth lead to Higher levels of oxidative stress

**Fig 3:**
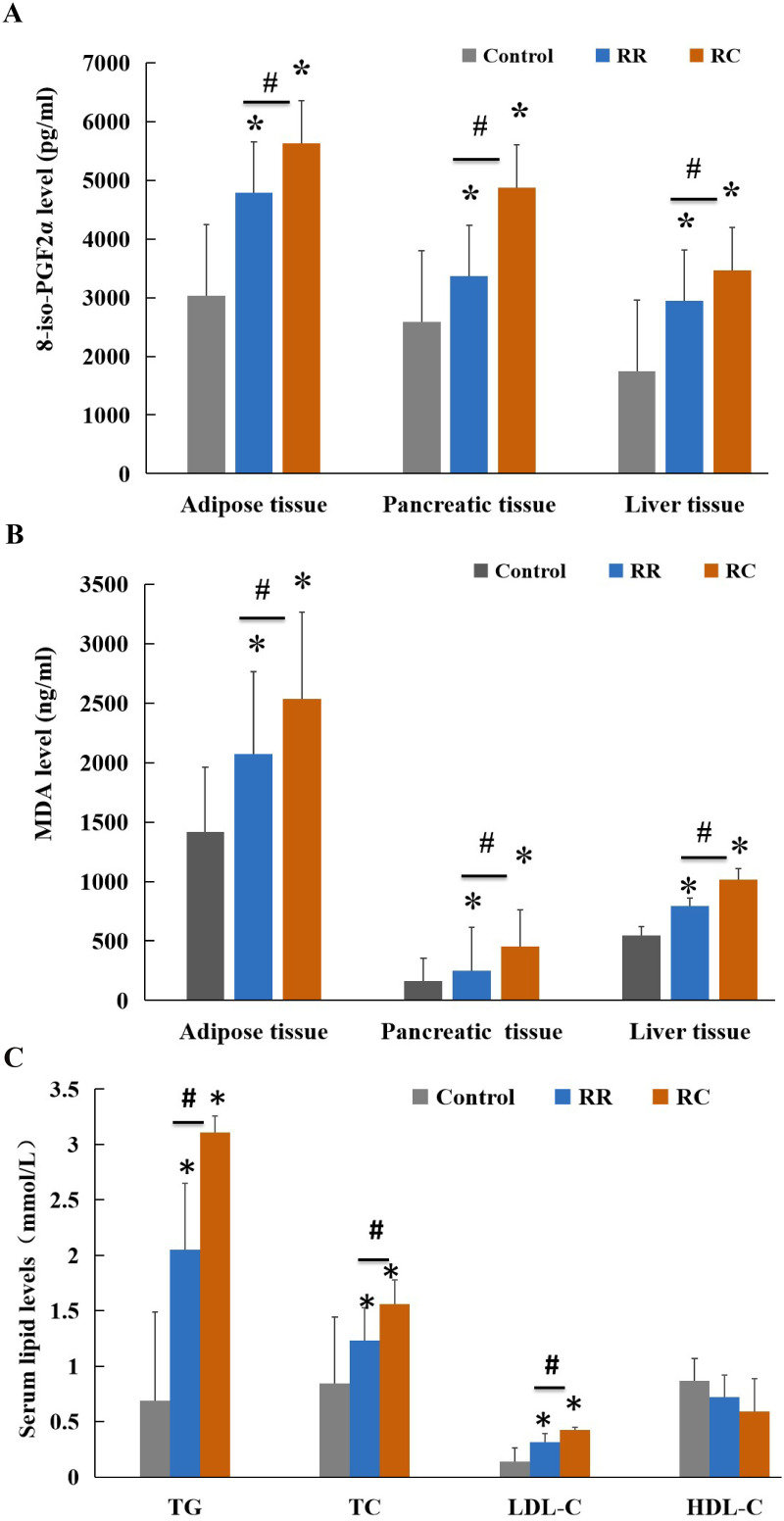
8-iso-PGF2α, MDA levels and Serum lipid levels increased in RC group. 8-iso-PGF2α **(A)** and MDA **(B)** levels in the adipose, pancreatic, and liver tissues of different groups of rats. 8-iso-PGF2α, a stable and specific marker for oxidative stress, was significantly increased in the RC group compared to the control and RR groups. **C**.Serum TC, TG, LDL-C, and HDL-C levels in different groups of rats at 9 months of age after birth.Control, non-IUGR rats receiving a normal diet during lactation and after weaning; RC, IUGR rats cross-fostering to a normal nutrition dam during lactation and receiving a normal diet after weaning; RR, IUGR rats receiving a low-protein diet during lactation and a normal diet after weaning; IUGR, intrauterine growth retardation; TC, total cholesterol; TG, triglyceride; LDL-C, low-density lipoprotein C; HDL-C, high-density lipoprotein C.*P < 0.05 vs Control; #P < 0.05 vs RR.

We measured the oxidative stress levels of the rats at 9 months of age. Levels of 8-iso-PGF2α and malondialdehyde (MDA) are considered indirect indices for evaluating oxidative stress [29]. Compared with the control group, levels of 8-iso-PGF2α in adipose, pancreatic, and liver tissues were increased in both the RC and RR groups (Fig 3A). In addition, 8-iso-PGF2α levels in adipose, pancreatic, and liver tissues were markedly higher in the RC group than in the RR group (P < 0.05). Adipose, pancreatic, and liver tissue MDA levels in the control group were significantly lower than those in the other two groups (Fig 3B). Compared with the RR group, the RC group exhibited higher MDA levels in adipose, pancreatic, and liver tissues. Our data demonstrated that IUGR offspring suffered from increased oxidative stress compared to non-IUGR controls. The adipose, pancreatic, and liver tissues of IUGR rats with rapid early catch-up growth were exposed to higher levels of oxidative stress.

### Early and accelerated catch-up growth show higher serum lipid level

In rat offspring, we assessed serum lipids, including serum TG, TC, LDL-C, and HDL-C, at 9 months of age after birth. Serum TG, TC, and LDL-C levels were higher in the RC group than in the control group; however, HDL-C levels were similar between the groups (Fig 3C). Further analysis revealed that three indices (serum TG, TC, and LDL-C) were significantly higher in the RC group than in the RR group (P < 0.05). These findings imply that blood lipid metabolic disorders occurred in IUGR offspring, especially in the later stage of early catch-up growth.

### Increased Sirtuin3 expression in pancreas and liver tissues of Early catch-up growth of IUGR offsprings

**Fig 4:**
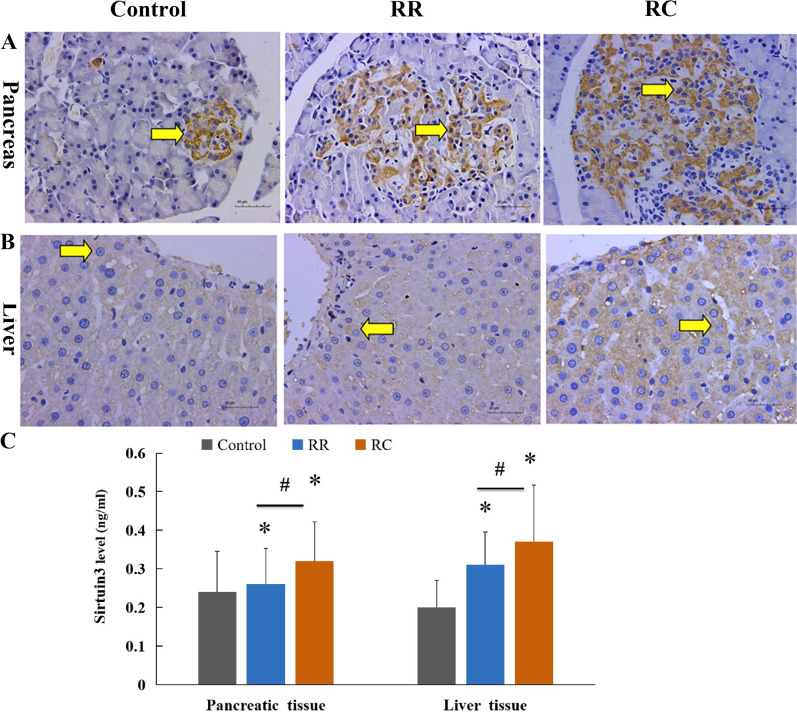
Expressions of Sirtuin3 obviously increased in RC group. **A.** IHC staining of sirtuin3 (yellow brown) in the cytoplasm of rat pancreatic cells. **B.** IHC staining of sirtuin3 (yellow brown) in the cytoplasm of rat liver cells.**C.** The quantification of the expression of Sirtuin3 in rats pancreas and liver.IHC, immunohistochemical staining; Control, non-IUGR rats receiving a normal diet during lactation and after weaning; RC, IUGR rats cross-fostering to a normal nutrition dam during lactation and receiving a normal diet after weaning;RR, IUGR rats receiving a low-protein diet during lactation and a normal diet after weaning;IUGR, intrauterine growth retardation. Scale bars, 40 µm. *P < 0.05 vs Control; #P < 0.05 vs RR.

Causal connections exist among ROS, lipid peroxidation, aging processes, and cell senescence [18]. Cellular senescence plays a physiological role during development. Sirtuins are a family of cellular sensors that exert a deacetylase function that regulates cellular bioenergetics by synchronizing nuclear and mitochondrial activities [30]. Among the seven sirtuins, including Sirtuin1-Sirtuin7, Sirtuin3 is expressed at relatively high levels in tissues with high oxidative capacity [31]. The protein content of Sirtuin3 is a metabolic balance sensor. In our study, Sirtuin3 was expressed mainly in the cytoplasmic region of the pancreas and liver tissues (Fig 4A). At 9 months of age after birth, the density of Sirtuin3 in the pancreas was significantly lower in the control group than in the RR and RC groups (P < 0.05, Fig 4A-B). The level of Sirtuin3 in the pancreatic tissues was higher in the RC group than in the RR group (P < 0.05). In liver tissues, Sirtuin3 protein expression level in the RC and RR groups was also significantly higher than that in the control group, and that in the RC group was even higher than that in the RR group (P < 0.05, Fig 4A-B). These findings imply that senescence may be involved in IUGR offspring with early catch-up growth.

### Mitochondrial TERT in pancreas and liver of IUGR with early catch-up growth

**Fig 5:**
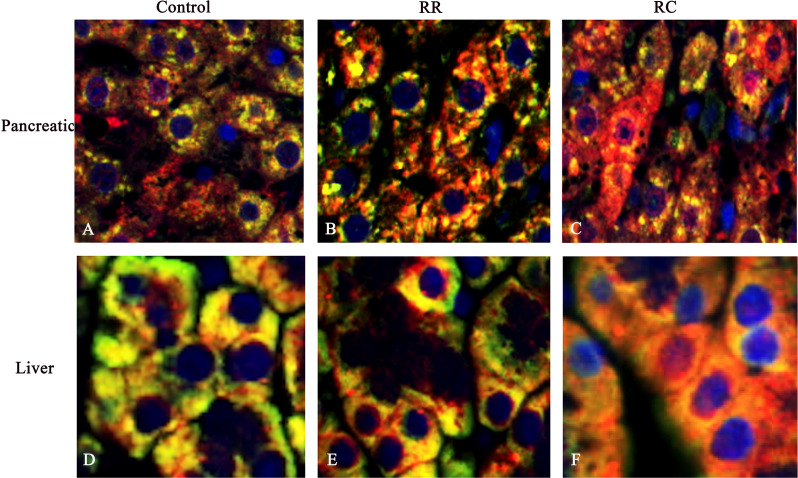
TERT shifted from nucleus to mitochondria in the pancreatic and liver cells of RC group. Immunofluorescence images with TERT (red), nuclear stain (DAPI, blue), and mitochondrial stain (green) in rat pancreas and liver cells. Note that in control offspring, TERT was detected in the cytoplasm and nucleus of rat pancreas **(A)** and liver cells **(D)**. In RR offspring, TERT was detected in the mitochondria of rat pancreatic cells **(B)** and liver cells **(E)**. In RC offspring, TERT was obviously shifted from the nucleus to the mitochondria in pancreatic **(C)** and liver tissues **(F)**. *Control, non-IUGR rats receiving a normal diet during lactation and after weaning; RC, IUGR rats cross-fostering to a normal nutrition dam during lactation and receiving a normal diet after weaning;RR, IUGR rats receiving a low-protein diet during lactation and a normal diet after weaning;IUGR, intrauterine growth retardation.Scale bars, 40 µm.*

TERT is found in the cytoplasm and mitochondria, along with its usual nuclear localization [32]. Previous studies have reported that oxidative stress can induce the accumulation of mitochondrial TERT by its translocation from the nucleus [33,34]. In this study, we used western blotting to determine whether mitochondrial TERT could be detected in pancreatic and liver tissues. Immunofluorescence co-localization revealed that TERT was localized in both the mitochondria and nuclei of pancreatic and liver tissues. In the Control group, TERT expression was observed mainly in the mitochondria (Fig 5A,D). The results showed that TERT was shifted from the nucleus to the mitochondria in the pancreatic and liver tissues of the RC group (Fig 5C,F), but not in the RR group (Fig 5B,E).

**Fig 6:**
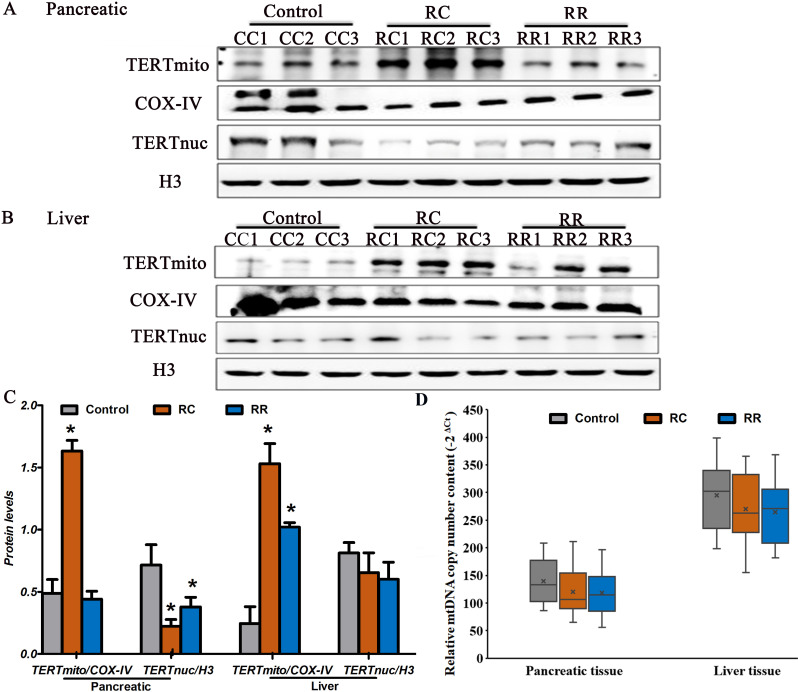
Protein levels of TERT in mitochondrial and nuclear increased in RC group. Note that in the pancreatic **(A)** and liver tissues **(B)** of RC offspring, TERT mitochondrial/COX-IV protein levels showed a significant increase compared to controls. **C.**The Quantification of the expression in rat pancreas and liver.**D.** Relative mtDNA content. The relative copy number of mtDNA was calculated by evaluating the ΔCt (2^-ΔCt^) of the reference gene. Data revealed that no significant differences in mtDNA content between RC group and Control group.Control, non-IUGR rats receiving a normal diet during lactation and after weaning; RC, IUGR rats cross-fostering to a normal nutrition dam during lactation and receiving a normal diet after weaning;RR, IUGR rats receiving a low-protein diet during lactation and a normal diet after weaning;IUGR, intrauterine growth retardation.*P < 0.05 vs Control.

In the pancreatic tissues of RC offspring, TERT mitochondrial/COX-IV protein levels showed a significant increase compared to the pancreas of controls (Fig 6A,C). The TERT nuc/H3 protein levels showed a decrease compared to those in the pancreas from controls. The ratio of mitochondrial/nuclear TERT in the pancreas was significantly elevated in the RC group compared with controls. Furthermore, the ratio of mitochondrial/nuclear TERT in the liver also increased in the RC group (Fig 6B,C). However, in the pancreatic and liver tissues of RR offspring, TERT mitochondrial/COX-IV protein levels also showed a slight increase compared to controls, suggesting that there is increased mitochondrial translocation of TERT under conditions of elevated oxidative stress in RC.

### The mtNDA content result

mtDNA content is an indicator of mitochondrial density and function. Previous studies have suggested that TERT protects mtDNA from oxidative damage. To determine the correlation between TERT and mtDNA content, RT-PCR analysis was conducted to assess the relative mtDNA content. The relative copy number of mtDNA was calculated by evaluating the ΔCt (2-ΔCt) of the reference gene. The data revealed no significant differences in the mtDNA content between the RC and control groups (Fig 6D, P > 0.05). Moreover, Spearman’s correlation test showed that the ratio of mitochondrial/nuclear TERT in pancreatic and liver tissues correlated positively with mtDNA content in the RC group. These data suggest a positive relationship between mtDNA content and mitochondrial TERT in pancreatic and liver tissues, and that the induction of mitochondrial TERT in the RC group could protect against oxidative damage to mtDNA.

## Discussion

Maternal protein restriction comprises both nutrient restriction and fetal hypoxia and affects placental size and insufficiency. Furthermore, the IUGR rat model, induced by prenatal nutritional restriction, has become an accepted model. This model has also been proven to be a sensitive model for investigating the links between postnatal catch-up growth and later development of metabolic disorders. In our study, we found that:1. Following enhanced nutritional supplementation after birth, pups with IUGR exhibited catch-up growth, as reflected by increased weight and growth rate. RC offspring showed early and accelerated postnatal catch-up growth. 2. In the pancreatic and liver tissues of RC offspring, early and accelerated catch-up growth led to telomere shortening at 9 months of age. Increased mitochondrial translocation of TERT may be involved in this phenomenon; 3. Oxidative stress and lipid peroxidation are involved in the pathogenesis of IUGR with catch-up growth, which is characterized by deregulated reactive oxygen species (ROS) production and blood lipid metabolic disorders, followed by Sirtuin3 protein expression. However, the molecular mechanisms underlying catch-up growth remain unclear.

Our study focused on male offspring to eliminate confounding effects of sex hormones on lipid metabolism. This design aligns with prior IUGR studies demonstrating sex-dimorphic responses to postnatal catch-up growth, in which males exhibit higher susceptibility to metabolic dysregulation [35].While our data did not address female outcomes, emerging evidence suggests sex-specific adaptations in IUGR models. Ying et al. found that estrogen-mediated antioxidant pathways in females may attenuate oxidative stress [36]. Females with IUGR often show preserved mitochondrial function and less severe metabolic phenotypes than males [37]. Therefore, male offspring will be included in future studies. IUGR refers to poor growth during pregnancy, which is considered the consequence of a disturbed intrauterine environment. Early acceleration of growth following IUGR is an important risk factor for later development of metabolic syndromes, such as obesity, diabetes, and cardiovascular diseases [38,39]. The catch-up growth theory explains the causes of metabolic diseases among individuals with IUGR [12,40]. In this study, weight was monitored and the growth rate after birth was presented. We found that the mean BWs was significantly different between the control and IUGR offspring. However, IUGR rats fed with control foster mothers showed early and postnatal catch-up growth from the end of the first postnatal week to 9 months of age. The pathophysiology of IUGR is characterized by abnormal oxidative stress and lipid peroxidation,resulting in deregulated production of reactive oxygen species (ROS), leading to oxidative stress [41]. In our study, adipose, pancreas, and liver tissues of the RC group exhibited higher ROS levels at 9 months of age when exposed to higher oxidative stress. This suggests that IUGR offspring with early catch-up growth are exposed to chronic oxidative stress. Moreover, our findings revealed telomere shortening in RC rats at 9 months, but not at 3 months, supporting a cumulative oxidative stress model. This aligns with IUGR studies, where postnatal catch-up growth drives progressive telomere attrition through sustained oxidative damage [42]. This delayed effect mirrors human data linking early rapid growth to later telomere shortening. We propose that chronic oxidative stress gradually depletes compensatory mechanisms (e.g., mitochondrial TERT translocation), ultimately overwhelming nuclear telomere maintenance. This temporal pattern underscores the critical role of the long-term oxidative burden in developmental metabolic programming. In addition, to assess whether lipid metabolic disorders developed in IUGR offspring, we measured four lipid metabolic indices. Increased TG,TC, and LDL-C levels at 9 months suggested abnormalities in lipid metabolism in IUGR offspring. The decreased LDL-C level further demonstrated that lipid metabolic disorders occurred during the later development of IUGR offspring. Based on these results, the precise effect and mechanism of postnatal dietary nutritional status on ROS and lipid metabolism disorders in IUGR offspring remains unknown.

Excessive ROS production may lead to a consequence of telomere shortening [43,44]. Previous studies have demonstrated that rapid postnatal catch-up growth may result in increased telomere shortening in tissues [18,42]. Telomerase is specifically designed as a reverse transcriptase and is a key factor for resisting telomere shortening. TERT, the main component of telomerase, serves as a template for telomere extension [45]. Recent studies have indicated that TL and telomerase activity are affected by rapid postnatal catch-up growth in various tissues [18]. In accordance with a previous study, we detected decreased TL length in the liver and pancreatic tissues of IUGR offspring at 9 months, which may be due to downregulated telomerase activity levels or the redistribution of telomerase out of the nucleus in stressed tissues. TERT is found in the cytoplasm and mitochondria, along with its usual nuclear localization. As much as 10–20% of total TERT is localized in the mitochondria and binds to mtDNA [46]. The results showed that TERT was shifted from the nucleus to the mitochondria in the pancreatic and liver tissues of RC group.There is increased mitochondrial translocation of TERT in under conditions of elevated oxidative stress in RC. Moreover, TERT binds to mtDNA and improves respiratory chain activity, protecting the mitochondria from environmental damage and decreasing reactive oxygen species (mtROS) production, which leads to mitochondrial damage and telomere shortening. Furthermore, we detected no changes in the mtDNA content in the RC group compared to that in the control group. It protects the mitochondria from oxidative stress damage. Our findings suggest a dual role for TERT under chronic oxidative stress in RC rats: **1) Mitochondrial Protection**: We observed that mitochondrial TERT translocation correlated with preserved mtDNA content (Fig 6D), despite elevated oxidative stress (Fig 3A-B). This aligns with previous studies showing that mitochondrial TERT binds to mtDNA, enhances respiratory chain activity, and reduces mitochondrial ROS (mtROS) production [23,47]. In our model, mitochondrial TERT likely acted as a compensatory mechanism to mitigate oxidative damage to mtDNA, thereby maintaining mitochondrial function. This is supported by the absence of significant mtDNA loss in RC rats compared to controls. **2) Nuclear Telomere Instability**: While mitochondrial TERT may protect mtDNA, nuclear telomere shortening in RC rats (Table 1) suggests that TERT redistribution reduces its availability for nuclear telomere maintenance. This is consistent with reports that oxidative stress downregulates nuclear telomerase activity and accelerates telomere attrition [44]. We propose that chronic oxidative stress creates a “trade-off” where TERT prioritizes mitochondrial protection over nuclear telomere elongation, exacerbating nuclear genomic instability. This imbalance may predispose the tissues to senescence or dysfunction later in life.

The present study provides valuable insights into the metabolic consequences of postnatal catch-up growth in IUGR offspring; however, several limitations should be acknowledged. Firstly, the study exclusively focused on male offspring to eliminate sex hormone confounders, yet sex-specific differences in metabolic programming are well-documented. Future studies should include female cohorts to elucidate potential sex dimorphisms in the developmental origins of metabolic diseases. Secondly, the follow-up period extended only to 9 months (equivalent to early adulthood in humans), leaving the long-term effects of accelerated catch-up growth on aging-related outcomes (e.g., lifespan, organ dysfunction) unexplored. Thirdly, while the study identified mitochondrial TERT translocation as a compensatory mechanism, the molecular regulators mediating this process (e.g., specific kinases, epigenetic modifications) remain unclear. So, the further study is need.

## Conclusion

Taken together, these data indicate that IUGR with early and rapid catch-up growth is exposed to chronic oxidative stress and subsequently affects TL and TERT translocation. Chronic oxidative stress may promote the translocation of TERT from the nucleus to mitochondria and protect tissues from oxidative stress damage. Our data support the hypothesis that impaired telomere homeostasis may play a role in the pathophysiology of IUGR with early catch-up growth, which may in turn predispose to metabolic disease later in life.

## Supporting information

S1_raw_images-TERTmito The original blot images of TERTmito in the pancreatic tissues. Note that protein bands in the original image. Each band is labeled with numbers 1–9, representing distinct experimental groups.*CC1(1),CC2(2),CC3(3): non-IUGR rats receiving a normal diet during lactation and after weaning; RC1,RC2,RC3: IUGR rats cross-fostering to a normal nutrition dam during lactation and receiving a normal diet after weaning;RR1,RR2,RR3: IUGR rats receiving a low-protein diet during lactation and a normal diet after weaning;IUGR, intrauterine growth retardation.*
(TIF)

S1_raw_images-COX-IV The original blot images of COX-IV in the pancreatic tissues. Note that protein bands in the original image. Each band is labeled with numbers 1–9, representing distinct experimental groups.*CC1(1),CC2(2),CC3(3): non-IUGR rats receiving a normal diet during lactation and after weaning; RC1,RC2,RC3: IUGR rats cross-fostering to a normal nutrition dam during lactation and receiving a normal diet after weaning;RR1,RR2,RR3: IUGR rats receiving a low-protein diet during lactation and a normal diet after weaning;IUGR, intrauterine growth retardation.*
(JPG)

S1_raw_images-TERTnuc The original blot images of TERTnuc in the pancreatic tissues. Note that protein bands in the original image. Each band is labeled with numbers 1–9, representing distinct experimental groups.*CC1(1),CC2(2),CC3(3): non-IUGR rats receiving a normal diet during lactation and after weaning; RC1,RC2,RC3: IUGR rats cross-fostering to a normal nutrition dam during lactation and receiving a normal diet after weaning;RR1,RR2,RR3: IUGR rats receiving a low-protein diet during lactation and a normal diet after weaning;IUGR, intrauterine growth retardation.*
(TIF)

S1_raw_images-H3 The original blot images of H3 in the pancreatic tissues. Note that protein bands in the original image. Each band is labeled with numbers 1–9, representing distinct experimental groups.*CC1(1),CC2(2),CC3(3): non-IUGR rats receiving a normal diet during lactation and after weaning; RC1,RC2,RC3: IUGR rats cross-fostering to a normal nutrition dam during lactation and receiving a normal diet after weaning;RR1,RR2,RR3: IUGR rats receiving a low-protein diet during lactation and a normal diet after weaning;IUGR, intrauterine growth retardation.*
(TIF)

S1_raw_images-TERTmito-liver The original blot images of TERTmito in the liver tissues. Note that protein bands in the original image. Each band is labeled with numbers 1–9, representing distinct experimental groups.*CC1(1),CC2(2),CC3(3): non-IUGR rats receiving a normal diet during lactation and after weaning; RC1,RC2,RC3: IUGR rats cross-fostering to a normal nutrition dam during lactation and receiving a normal diet after weaning;RR1,RR2,RR3: IUGR rats receiving a low-protein diet during lactation and a normal diet after weaning;IUGR, intrauterine growth retardation.*
(TIF)

S1_raw_images-COX-IV-liver The original blot images of COX-IV in the liver tissues. Note that protein bands in the original image. Each band is labeled with numbers 1–9, representing distinct experimental groups.*CC1(1),CC2(2),CC3(3): non-IUGR rats receiving a normal diet during lactation and after weaning; RC1,RC2,RC3: IUGR rats cross-fostering to a normal nutrition dam during lactation and receiving a normal diet after weaning;RR1,RR2,RR3: IUGR rats receiving a low-protein diet during lactation and a normal diet after weaning;IUGR, intrauterine growth retardation.*
(JPG)

S1_raw_images-H3-liver The original blot images of H3 in the liver tissues. Note that protein bands in the original image. Each band is labeled with numbers 1–9, representing distinct experimental groups.*CC1(1),CC2(2),CC3(3): non-IUGR rats receiving a normal diet during lactation and after weaning; RC1,RC2,RC3: IUGR rats cross-fostering to a normal nutrition dam during lactation and receiving a normal diet after weaning;RR1,RR2,RR3: IUGR rats receiving a low-protein diet during lactation and a normal diet after weaning;IUGR, intrauterine growth retardation.*
(TIF)

S1_raw_images-TERTnuc-liver The original blot images of TERTnuc in the liver tissues. Note that protein bands in the original image. Each band is labeled with numbers 1–9, representing distinct experimental groups.*CC1(1),CC2(2),CC3(3): non-IUGR rats receiving a normal diet during lactation and after weaning; RC1,RC2,RC3: IUGR rats cross-fostering to a normal nutrition dam during lactation and receiving a normal diet after weaning;RR1,RR2,RR3: IUGR rats receiving a low-protein diet during lactation and a normal diet after weaning;IUGR, intrauterine growth retardation.*
(TIF)
