# Supplementary figures and images for: TERT translocation as a Novel condition in Intrauterine Growth Restriction rats with early catch-up growth
Source: PLoS One. 2025 Jun 5;20(6):e0312221. doi: 10.1371/journal.pone.0312221 (PMC12140425; doi:10.1371/journal.pone.0312221)

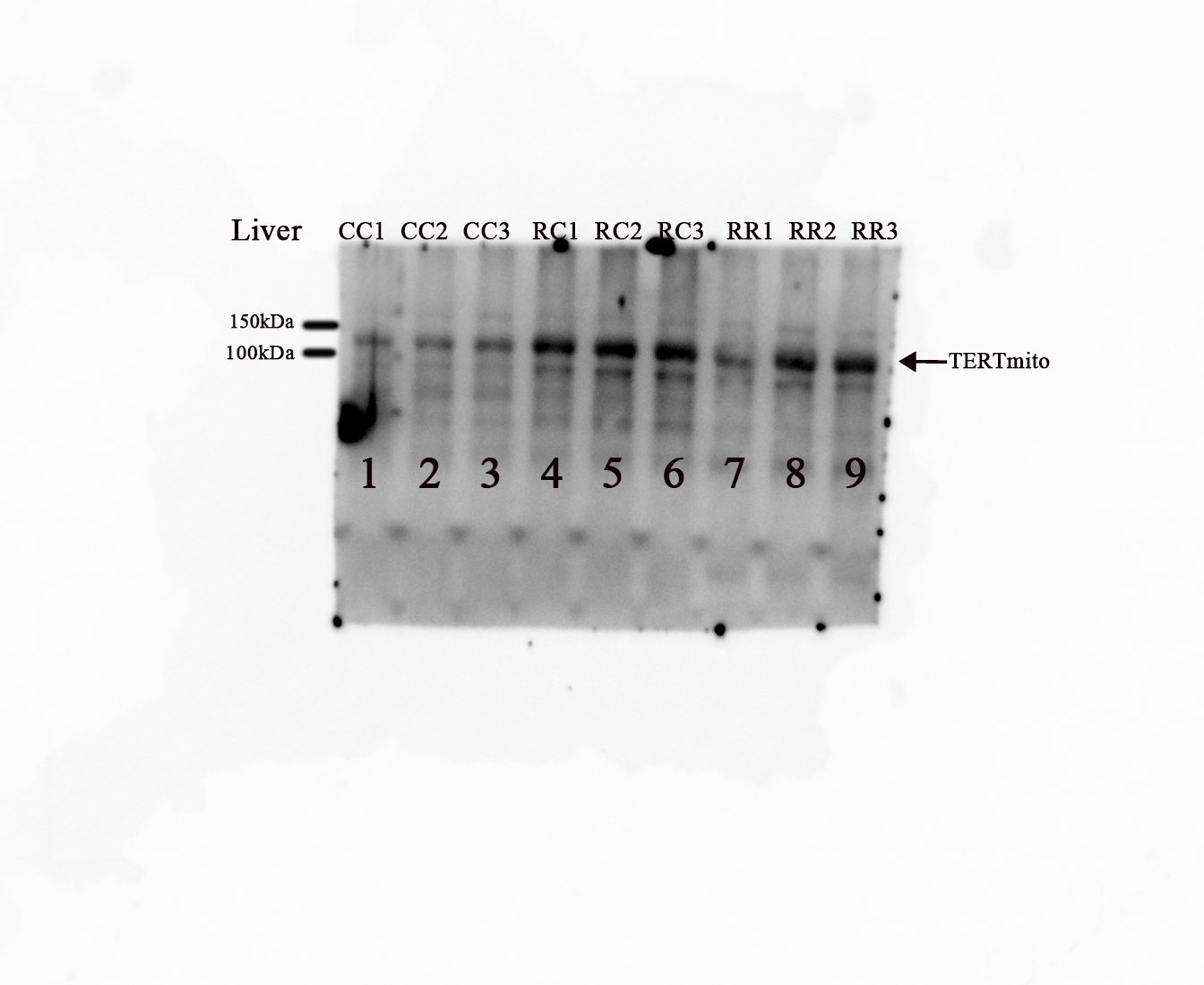

Supplement: S1_raw_images-TERTmito — Note that protein bands in the original image. Each band is labeled with numbers 1–9, representing distinct experimental groups.CC1(1),CC2(2),CC3(3): non-IUGR rats receiving a normal diet during lactation and after weaning; RC1,RC2,RC3: IUGR rats cross-fostering to a normal nutrition dam during lactation and receiving a normal diet after weaning;RR1,RR2,RR3: IUGR rats receiving a low-protein diet during lactation and a normal diet after weaning;IUGR, intrauterine growth retardation. (TIF) [file pone.0312221.s001.tif]

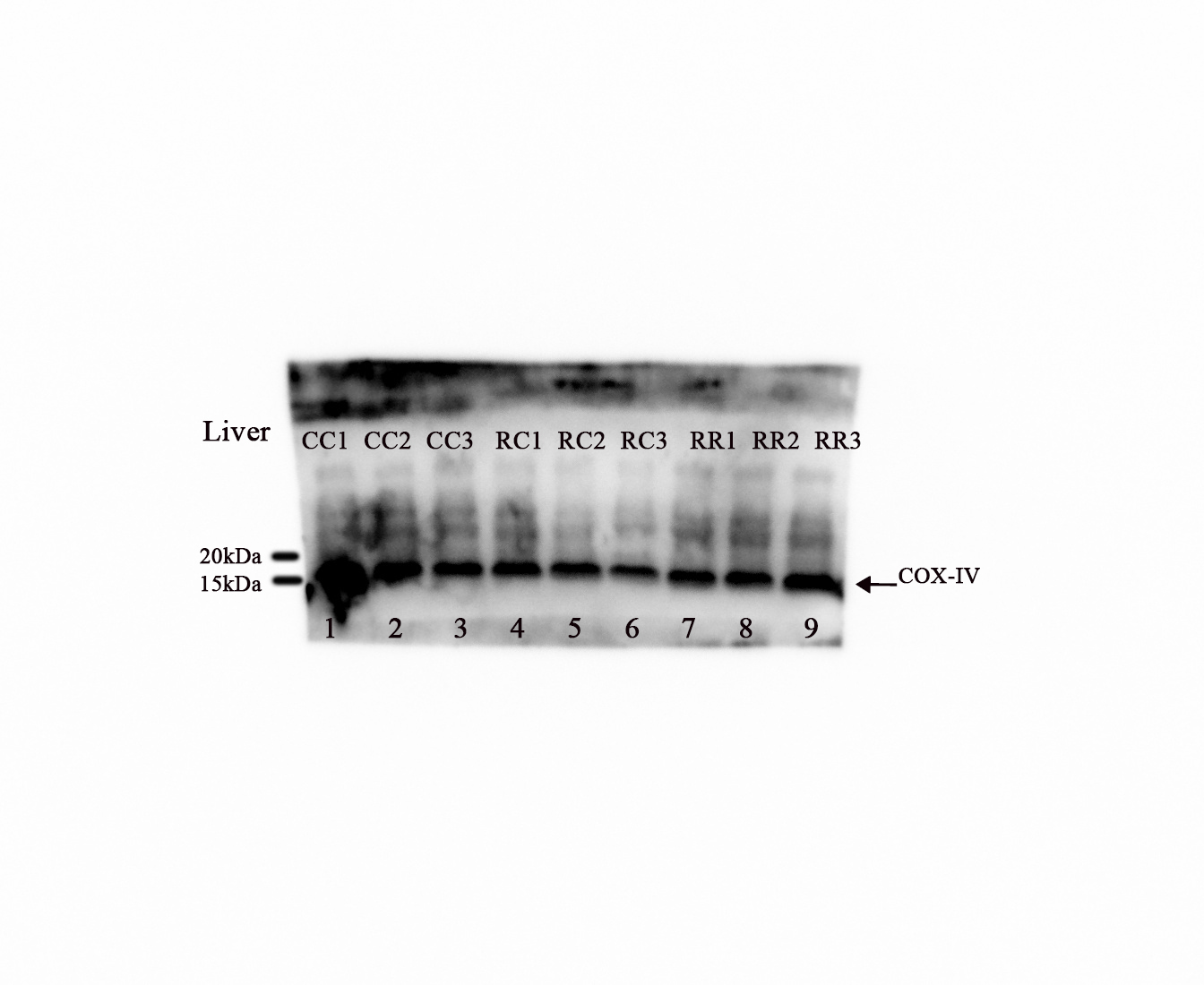

Supplement: S1_raw_images-COX-IV — Note that protein bands in the original image. Each band is labeled with numbers 1–9, representing distinct experimental groups.CC1(1),CC2(2),CC3(3): non-IUGR rats receiving a normal diet during lactation and after weaning; RC1,RC2,RC3: IUGR rats cross-fostering to a normal nutrition dam during lactation and receiving a normal diet after weaning;RR1,RR2,RR3: IUGR rats receiving a low-protein diet during lactation and a normal diet after weaning;IUGR, intrauterine growth retardation. (JPG) [file pone.0312221.s002.jpg]

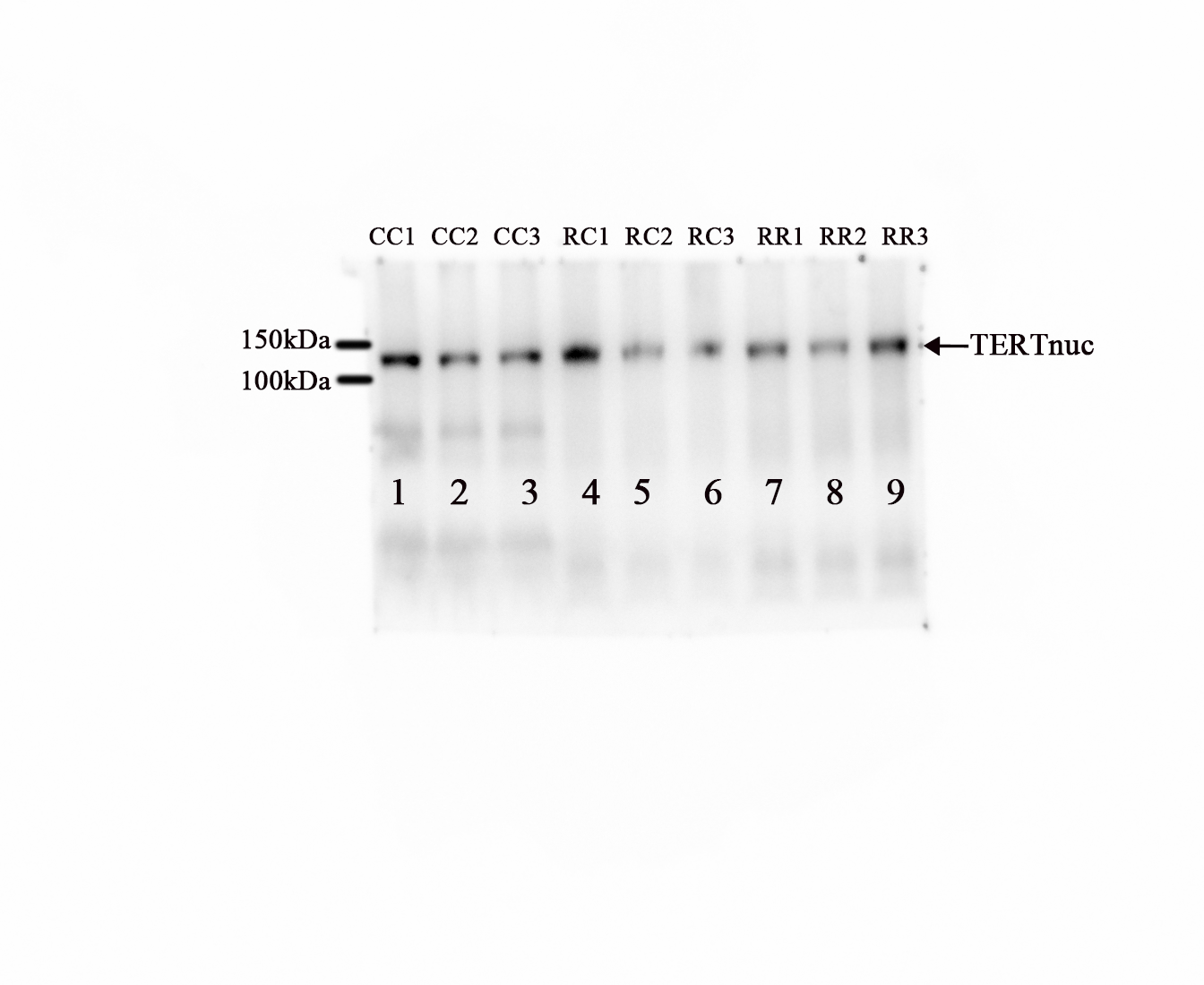

Supplement: S1_raw_images-TERTnuc — Note that protein bands in the original image. Each band is labeled with numbers 1–9, representing distinct experimental groups.CC1(1),CC2(2),CC3(3): non-IUGR rats receiving a normal diet during lactation and after weaning; RC1,RC2,RC3: IUGR rats cross-fostering to a normal nutrition dam during lactation and receiving a normal diet after weaning;RR1,RR2,RR3: IUGR rats receiving a low-protein diet during lactation and a normal diet after weaning;IUGR, intrauterine growth retardation. (TIF) [file pone.0312221.s003.tif]

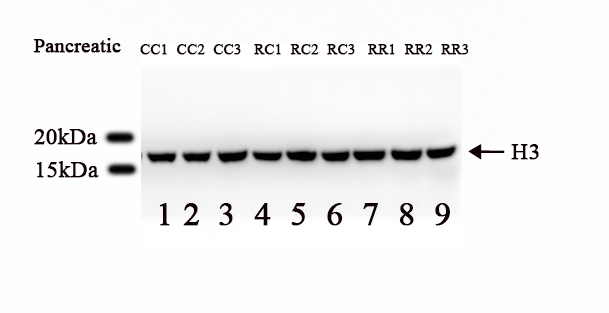

Supplement: S1_raw_images-H3 — Note that protein bands in the original image. Each band is labeled with numbers 1–9, representing distinct experimental groups.CC1(1),CC2(2),CC3(3): non-IUGR rats receiving a normal diet during lactation and after weaning; RC1,RC2,RC3: IUGR rats cross-fostering to a normal nutrition dam during lactation and receiving a normal diet after weaning;RR1,RR2,RR3: IUGR rats receiving a low-protein diet during lactation and a normal diet after weaning;IUGR, intrauterine growth retardation. (TIF) [file pone.0312221.s004.tif]

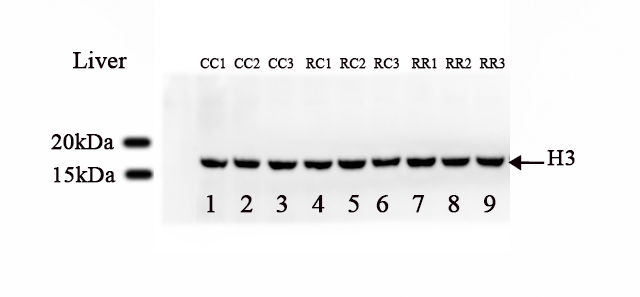

Supplement: S1_raw_images-H3-liver — Note that protein bands in the original image. Each band is labeled with numbers 1–9, representing distinct experimental groups.CC1(1),CC2(2),CC3(3): non-IUGR rats receiving a normal diet during lactation and after weaning; RC1,RC2,RC3: IUGR rats cross-fostering to a normal nutrition dam during lactation and receiving a normal diet after weaning;RR1,RR2,RR3: IUGR rats receiving a low-protein diet during lactation and a normal diet after weaning;IUGR, intrauterine growth retardation. (TIF) [file pone.0312221.s007.tif]
